# Targeting the PRMT1-cGAS-STING signaling pathway to enhance the anti-tumor therapeutic efficacy

**DOI:** 10.46439/cancerbiology.5.064

**Published:** 2024

**Authors:** Daoyuan Huang, Abdol-Hossein Rezaeian, Jingchao Wang, Yihang Qi, Hong Chen, Hiroyuki Inuzuka, Wenyi Wei

**Affiliations:** 1 Department of Pathology, Beth Israel Deaconess Medical Center, Harvard Medical School, Boston, MA 02215, USA; 2 Vascular Biology Program, Department of Surgery, Harvard Medical School, Boston Children’s Hospital, Boston, MA 02115, USA

## Abstract

Activating innate immune signaling in tumor cells to enhance anti-tumor immunity and increase T cell-mediated killing is the core objective of tumor immunotherapy. PRMT1, one of the most crucial PRMTs, plays a critical role in tumor progression and innate immunity. Recent research revealed that PRMT1 can inhibit the enzymatic activity of cGAS in part through PRMT1-mediated Arg methylation, thereby suppressing the anti-tumor immune response of cells. As such, inhibiting or knocking down PRMT1 can synergistically enhance the efficacy of anti-PD-1 immunotherapy by activating the cGAS-STING signaling pathway. Here, we provide a comprehensive description of the two key signaling components, PRMT1 and cGAS, in the PRMT1-cGAS-STING signaling pathway for therapeutic intervention to augment anti-tumor immunity. By understanding the specific physiological functions and regulatory mechanisms of PRMT1, as well as the extensive post-translational modifications (PTMs) of cGAS, we have identified several compounds and drugs that can directly target PRMT1 or cGAS, and/or indirectly target PRMT1 upstream regulators or cGAS-post-translational modifying enzymes as potential means to activate the cGAS-STING signaling pathway. However, further investigation is needed on the efficacy of combining this pathway activation with anti-PD1 therapy. This review suggests that targeting the PRMT1-cGAS-STING pathway with immune checkpoint inhibitors is likely a promising approach in tumor immunotherapy.

## Protein Arginine Methylation and PRMTs

**Figure 1.**
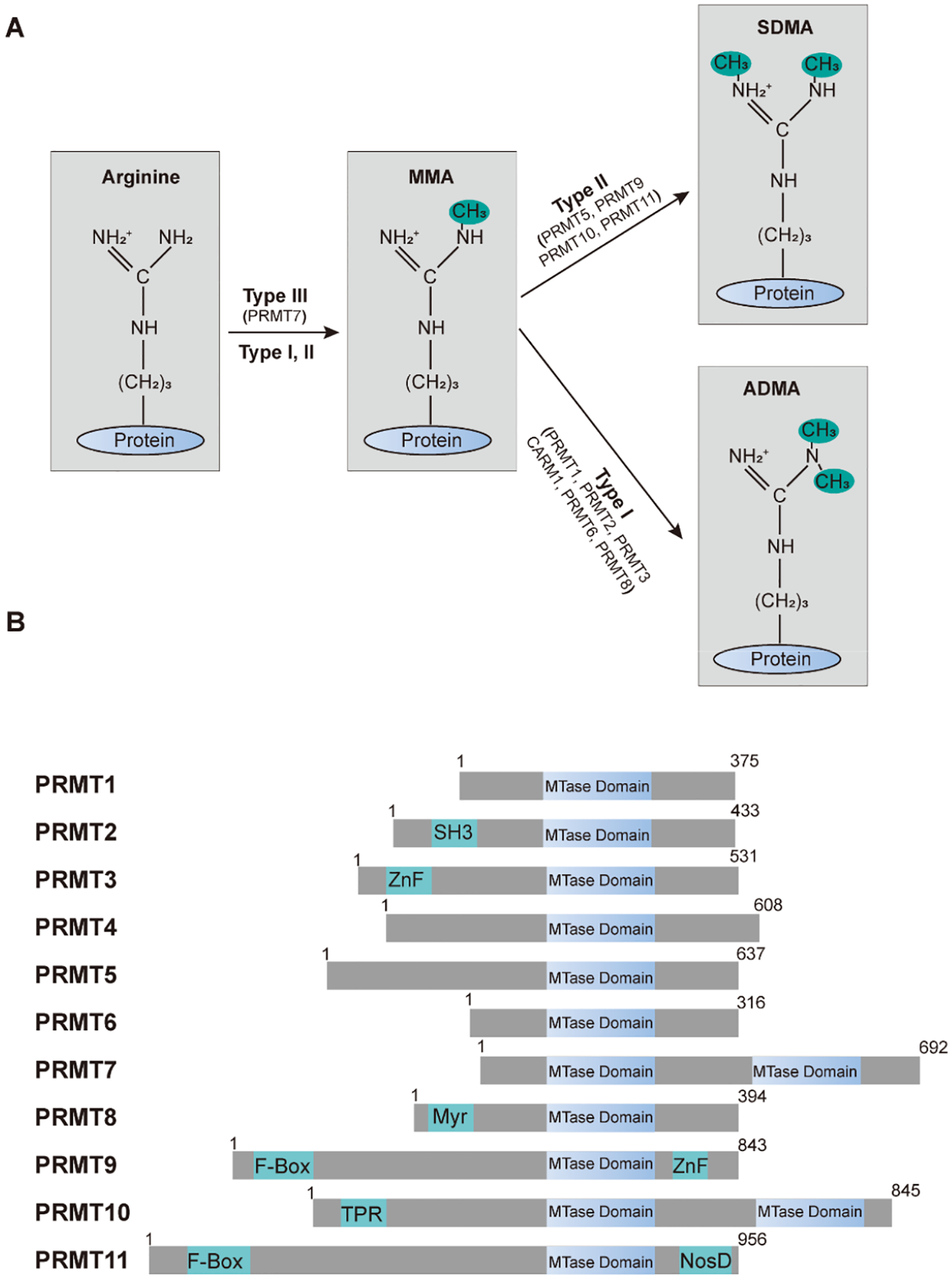
Protein arginine methylation and the family of PRMTs. **(A)** Arginine methyltransferases and their impact on protein arginine methylation patterns. The arginine residue possesses five potential hydrogen bond donors. In mammals, PRMTs utilize the methyl group from S-adenosyl-L-methionine (SAM) to create mono-methylarginine (MMA). Subsequently, type I PRMTs add an additional methyl group to the same nitrogen atom, resulting in asymmetric dimethylarginine (ADMA), whereas type II PRMTs produce symmetric dimethylarginine (SDMA). **(B)** A schematic illustration of the human PRMT family members. Every contains at least one conserved MTase domain with signature motifs I, post-I, II, and III, along with a THW loop. Additional domains are highlighted: SH3, ZnF (zinc finger), Myr (myristoylation), F-box, TPR (tetratricopeptide), and NosD (nitrous oxidase accessory protein).

Post-translational modifications of proteins alter their biophysical properties, affecting stability, localization, and interactions, which are essential for proteome diversity and cellular homeostasis [1,2]. Protein arginine methylation is a post-translational modification where methyl groups are added to the arginine residues of substrate proteins [3,4], typically catalyzed by protein arginine methyltransferases (PRMTs) [5]. This modification can occur as mono-methylation (MMA), asymmetric dimethylarginine (ADMA), or symmetric dimethylarginine (SDMA) [6,7] (Figure 1). Arginine methylation affects protein function, interactions, and localization, which is crucial in regulating gene expression, signal transduction, RNA processing, and DNA repair [5]. This modification is essential for cellular homeostasis and various biological processes.

Protein arginine methyltransferases (PRMTs) are the enzymes responsible for catalyzing the transfer of methyl groups to arginine residues on target proteins. In mammals, there are 11 known PRMTs [8,9], and they are classified into three types based on the methylation pattern they produce. Type I PRMTs: catalyze the formation of ADMA and MMA [8]. This group includes PRMT1, PRMT2, PRMT3, PRMT4 (also known as CARM1), PRMT6, and PRMT8 [8]. Type II PRMTs: catalyze the formation of SDMA and MMA [8]. This group includes PRMT5, PRMT9 (also known as FBXO11), PRMT10, and PRMT11 (also known as FBXO10). Type III PRMTs: catalyze only MMA. This group includes PRMT7 [8]. These enzymes share common features such as highly conserved methyltransferase domains (Figure 1B) and the use of S-adenosylmethionine (SAM) as the methyl donor, and they play crucial roles in various cellular processes, including signal transduction, gene regulation, and RNA metabolism [5,10–12].

## PRMT1 and Its Function

PRMT1 is the earliest discovered and most predominant protein arginine methyltransferase [13]. It regulates over 90% of arginine methylation in mammalian cells by catalyzing the methylation of arginine side chains, specifically forming MMA and ADMA [14]. Numerous studies have shown that PRMT1 primarily targets protein substrates with conserved glycine and arginine-rich GAR (RG/RGG/RXG) motifs, which are often located at the C-terminus of proteins [15–17].

PRMT1 is involved in various biological processes by modifying different types of downstream substrates, including, but not limited to, DNA damage repair, gene transcription regulation, and signal transduction abnormalities [18,19]. These activities influence various physiological and pathological processes such as cellular senescence [20], tumorigenesis [21], spermatogenesis [22], and muscle stem cell fate [23].

In the following sections, we will discuss PRMT1’s biological role in physiological processes, cancer, and innate immunity.

### PRMT1-mediated arginine methylation in physiological processes

PRMT1 is involved in many biological processes through the methylation of different substrates (Table 1). For instance, PRMT1 promotes H3R4 methylation to enhance β-globin transcription [24], regulates cellular senescence [20], and influences spermatogonial stem cell development [22]. It also facilitates DNA damage repair in part through promoting the methylation of 53BP1 [25,26] and MRE11 [25,27].

Furthermore, PRMT1 impacts gene transcription by methylating RUNX1 [28], STAT1 [29], MyoD [30], and TAF15 [31]. Moreover, PRMT1 controls ATXN2L localization by methylation [32]. Additionally, PRMT1 participates in various developmental and functional processes such as pancreatic endocrine development of hESCs [33], muscle stem cell fate determination [23], and the regulation of T cell and macrophage functions [34,35]. Our group found that PRMT1 methylates NPRL2, cooperating with SAMTOR to regulate mTORC1 sensing of methionine [36]. The ever-growing list of PRMT1 downstream substrates demonstrates the critical roles in various cellular processes, which is summarized in Table 1.

### The role of PRMT1-mediated arginine methylation in tumor progression

**Figure 2.**
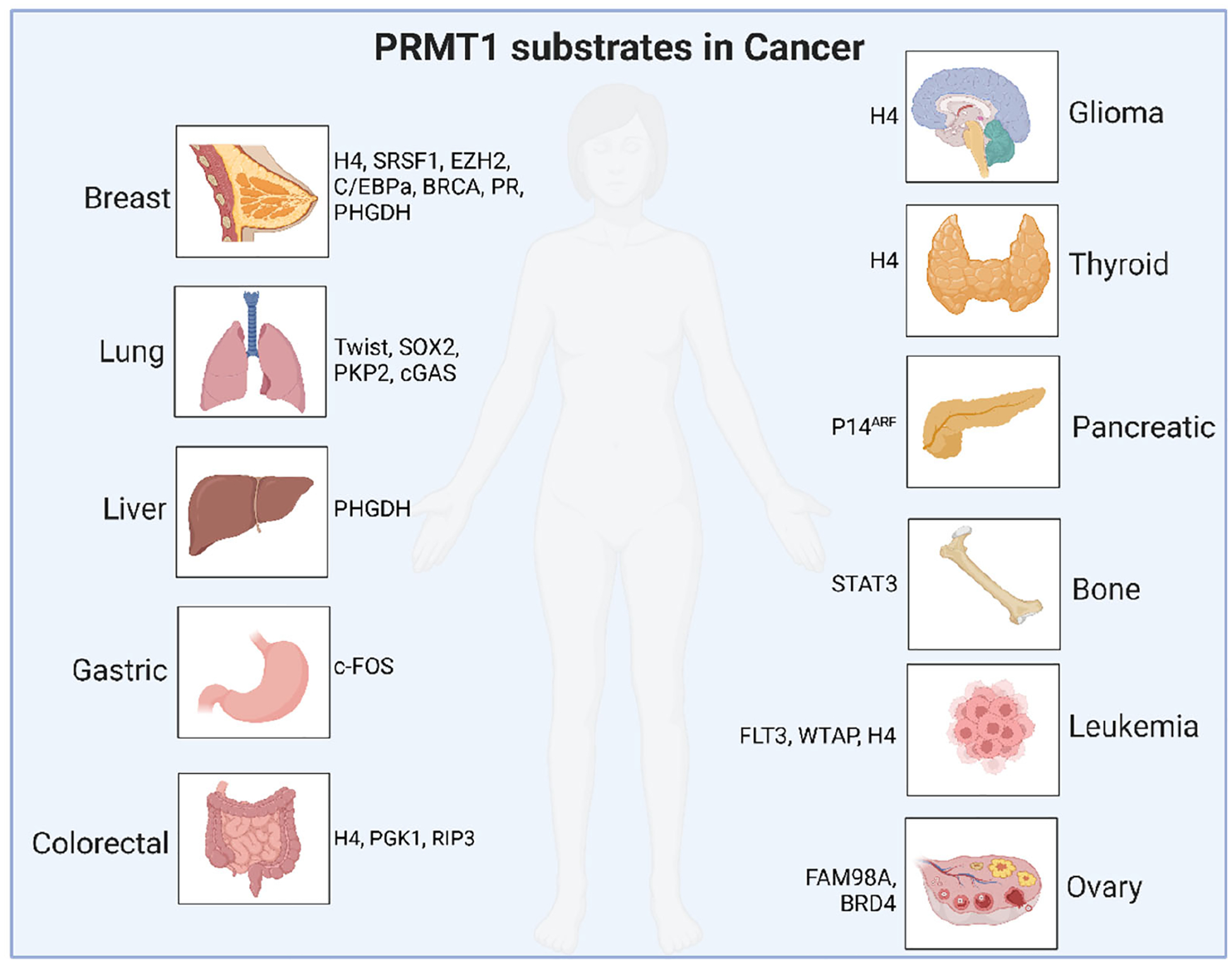
A schematic summary of PRMT1 downstream methylation substrates in various types of human cancers.

The functions and regulatory mechanisms of PRMT1 in cancer progression have been extensively studied to uncover potential PRMT1-dependent therapeutic strategies, and please refer to the review articles for details [19,21]. Here, we provide a comprehensive overview of the methylation substrates and functions of PRMT1 in various types of human tumors (Figure 2, Table 2) to explore its potential value in cancer therapy.

Notably, in breast cancer, PRMT1 promotes tumor cell proliferation, tumorigenesis, and metastasis by methylating substrates such as H4 [52], BRCA1 [53], C/EBPα [54], EZH2 [55–57], Progesterone Receptor (PR) [58], PHGDH [59], and SRSF1 [60]. In lung cancer, PRMT1 methylates and stabilizes cGAS protein levels, thereby promoting NSCLC proliferation [61]; it also methylates TWIST [62] to regulate EMT and methylates PKP2 [63] and SOX2 [64], leading to radioresistance and chemoresistance. In colorectal cancer, the methylation of H4 [65], PGK1 [66], and RIP3 [67] regulates tumor progression and immune evasion. Previously, we found that PRMT1 promotes tumorigenesis by regulating m6A modification levels in part through promoting the methylation of METTL14 at both R442 and R445 residues [68]. PRMT1 is also involved in the progression of the liver [69], osteosarcoma [70], glioma [71], thyroid, pancreatic [72], leukemia [73–76], ovarian [77,78], and gastric [79] cancers by methylating various substrate proteins as summarized in Table 2. Due to space limits, we will not elaborate on these Arg methylation events and their biological functions here.

### The possible role of PRMT1 in innate immunity

In addition to its involvement in numerous physiological processes and tumor progression through the methylation of various substrates, PRMT1 also plays a crucial role in innate immunity. Studies using CRISPR screening have identified PRMT1 as a negative regulator of CD8+ T cells. Mechanistically, PRMT1 suppresses STAT1, thereby downregulating interferon-gamma (IFN-γ)-induced MHC-I expression [83]. Additionally, Class II transactivator (CIITA) mediates MHC-II expression through IFN-γ. PRMT1 has been found to methylate CIITA, promoting its degradation and thus inhibiting MHC-II transcription [84].

Notably, inhibition of PRMT1 can modulate the enhancer region of DNMT1, increasing levels of H4R3me2a and H3K27ac, which downregulates DNMT1 expression and activates endogenous retrovirus (ERV) transcription and interferon signaling [85]. Besides, our study revealed that PRMT1 methylates the Arg133 residue of the cGAS protein, subsequently preventing its dimerization to inhibit the cGAS-STING pathway [86].

These studies collectively demonstrate that PRMT1 is likely a negative regulator of tumor immunity. Hence, genetic or pharmacological inhibition of PRMT1 can activate type I and type II interferon expression, thereby enhancing the efficacy of anti-PD-1 immunotherapy.

### Upstream regulation of PRMT1

Given the crucial role PRMT1 plays in both physiological and pathological conditions, a comprehensive understanding of its regulatory mechanisms is of significant importance. Current research on PRMT1 regulation mainly focuses on upstream pathways that can impact its expression levels and enzymatic activity (Table 3). In LO2 cells, fasting reduces PRMT1 expression via the mTOR-p-STAT signaling pathway, suggesting that mTOR acts as a positive regulator of PRMT1 expression [87]. Additionally, BTG1 serves as a methylation coactivator for PRMT1, enhancing its methylation of ATF4 [88]. Conversely, several mechanisms were found to negatively regulate PRMT1. To this end, PP2A [89], TR3 [90], and hCAF1 [91] inhibit PRMT1’s enzymatic activity through direct interaction. Three E3 ubiquitin ligases—E4B [92], CHIP [92,93], and TRIM48 [94]—promote PRMT1 degradation via ubiquitination. Deferoxamine (DFO)/iron deficiency and miR-503 downregulate PRMT1 mRNA levels [95,96]. Despite these known regulators and mechanisms, further research is needed to explore other post-translational modifications of PRMT1, such as phosphorylation, acetylation, methylation, and palmitoylation, and their effects on PRMT1’s function.

## An Overview of the cGAS-STING Pathway

**Figure 3.**
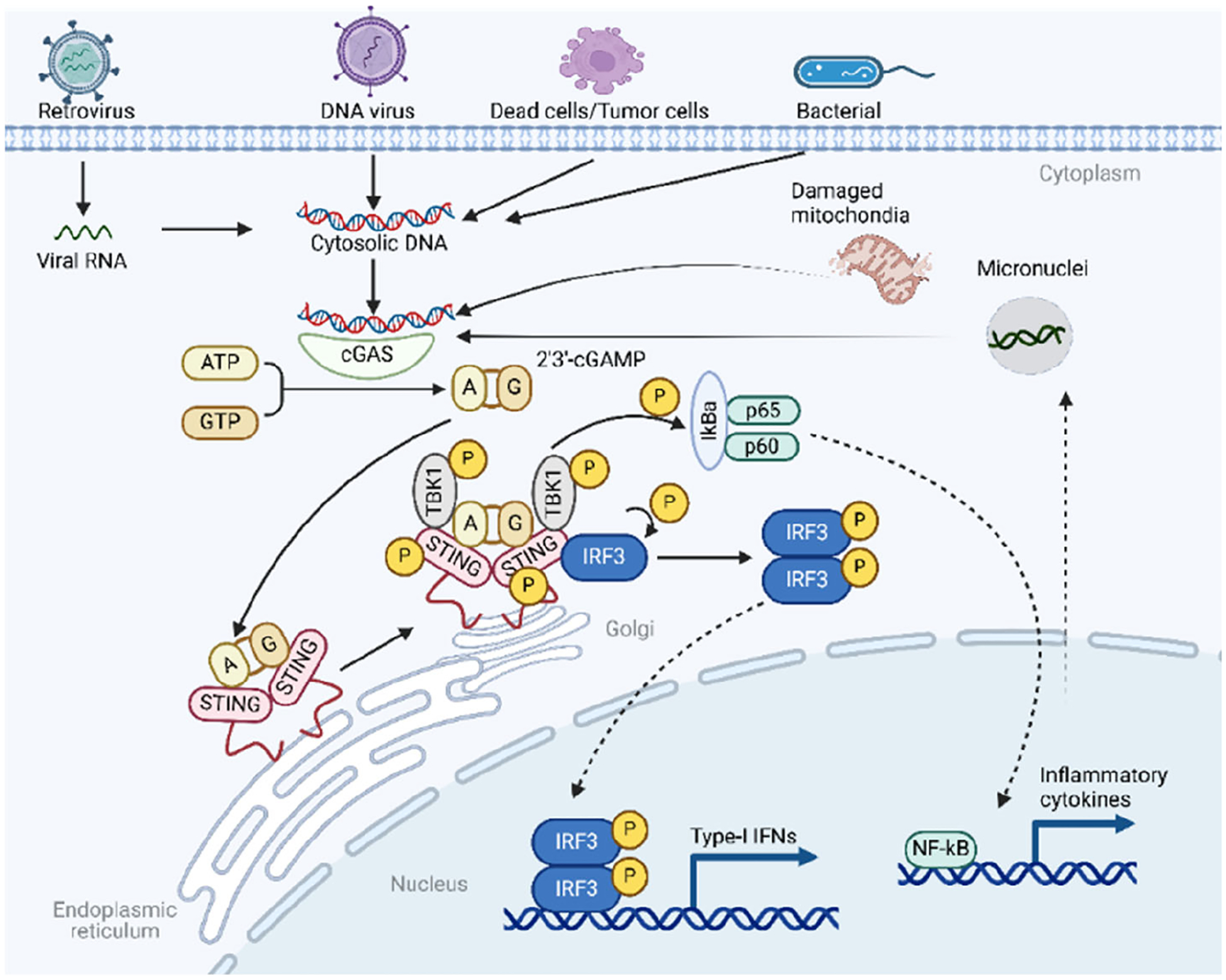
A schematic overview of the cGAS-STING-TBK1 signaling pathway.

The cGAS-STING pathway is a crucial component of the innate immune system, responsible for detecting cytosolic double-stranded DNA (dsDNA), which indicates infections, cellular damage, or cancer [97]. The pathway initiates when cGAS (cyclic GMP-AMP synthase) binds to dsDNA in the cytoplasm [98]. This binding activates cGAS, allowing it to synthesize cyclic 2’3’-GMP-AMP (cGAMP) from ATP and GTP. The cGAMP acts as a second messenger, binding to the STING (Stimulator of Interferon Genes) protein on the endoplasmic reticulum membrane [98]. This binding induces a conformational change in STING, leading to its activation and translocation to the Golgi apparatus and perinuclear vesicles. Activated STING then recruits TANK-binding kinase 1 (TBK1), which plays a crucial role in downstream signaling [99]. TBK1 phosphorylates interferon regulatory factor 3 (IRF3), causing IRF3 to dimerize and translocate to the nucleus [100]. In the nucleus, IRF3, along with other transcription factors like NF-κB, induces the expression of type I interferons (e.g., IFN-β) and inflammatory cytokines [100]. These molecules are essential for mounting an effective antiviral response and modulating the immune system to address the presence of cytosolic DNA (Figure 3). Through this cascade, the cGAS-STING pathway effectively translates the detection of abnormal cytosolic DNA into a potent immune response, underscoring its importance in antiviral defense, cancer immunity, and its potential role in autoimmune conditions when dysregulated.

## The Post-Translational Modifications (PTMs) of cGAS

**Figure 4.**
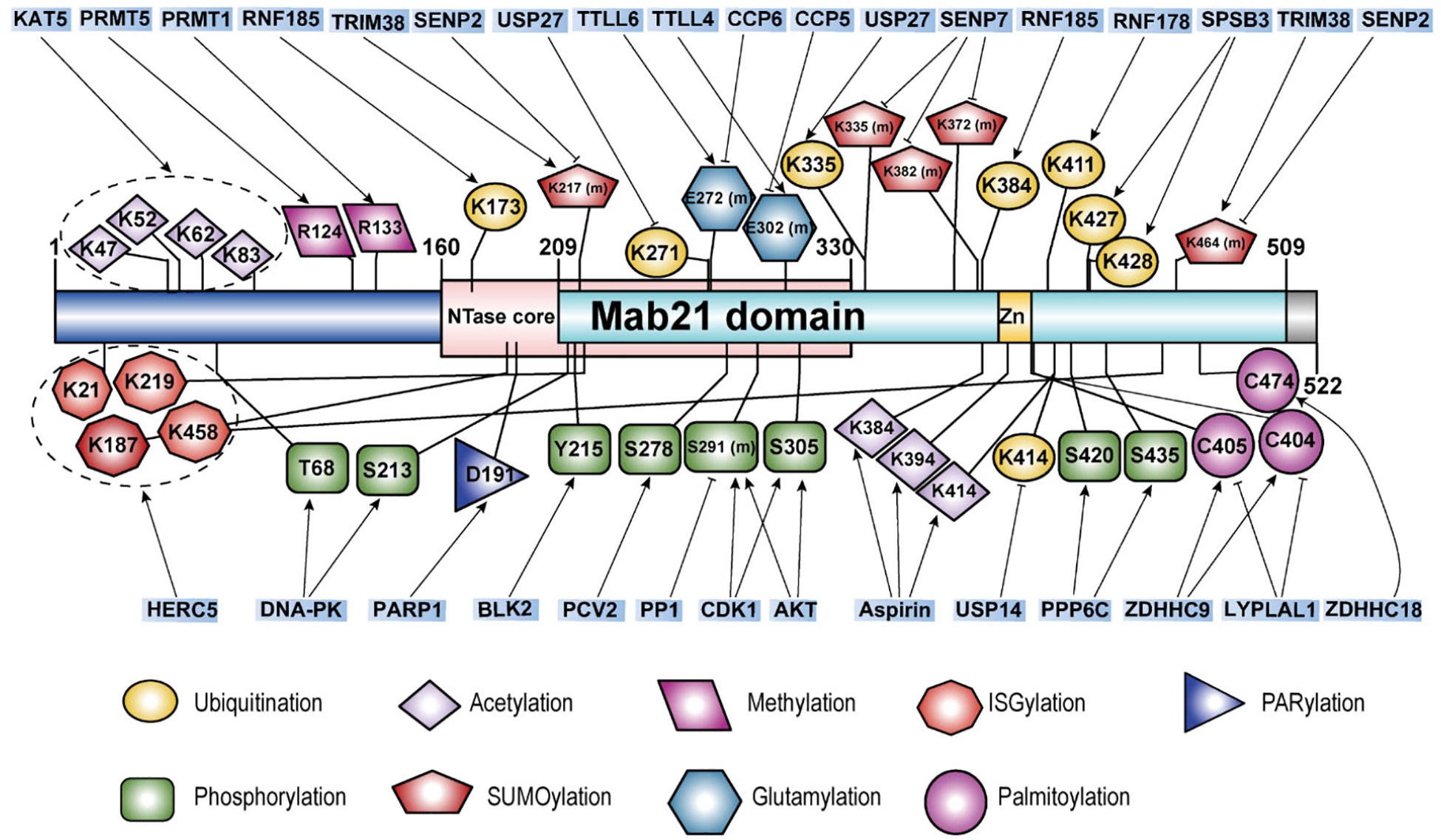
A schematic summary of the known protein post-translational modifications (PTMs) of cGAS.

Accurate recognition of immunostimulatory DNA by cGAS is crucial for the proper activation of the innate immune response. The post-translational modifications (PTMs) of the cGAS protein are essential for regulating its enzymatic activity and maintaining immune homeostasis under both physiological and pathological conditions. Below, we summarize the known PTMs of cGAS, including ubiquitination (both degradative and non-degradative), phosphorylation, acetylation, SUMOylation, methylation, glutamylation, ISGylation, palmitoylation, and PARylation (Figure 4, Table 4). These PTMs influence various aspects of cGAS cellular function by regulating its enzyme activity, DNA binding, protein stability, and subcellular localization.

### Ubiquitination

Ubiquitination is a critical post-translational modification that involves the covalent attachment of ubiquitin to a target protein. This process affects the protein’s stability, interactions, and biological functions and plays a role in cell survival, differentiation, and the regulation of both innate and adaptive immunity [101]. Ubiquitination plays a significant role in regulating cGAS through degradation mechanisms. The Hepatitis B virus X protein (HBx) suppresses IFN-I production by directly promoting the ubiquitination and autophagic degradation of cGAS [102]. Furthermore, the CRL5-SPSB3 ubiquitin ligase targets nuclear cGAS for degradation, highlighting the importance of ubiquitination in controlling cGAS levels and activity [103]. Ubiquitination of cGAS also involves non-degradative regulatory mechanisms. Various ubiquitin ligases activate cGAS by mediating different forms of ubiquitin chains, such as TRAF6 (undefined) [104], RNF185 (K27) [105], TRIM41 (mono) [106], and TRIM56 (mono) [107]. Conversely, RNF178 mediates K63-linked ubiquitination, which inhibits the DNA-binding ability of cGAS [108]. Deubiquitination, the process of removing ubiquitin from target proteins, often counteracts the effects of ubiquitination [109,110]. Deubiquitinases like USP14 [111], USP27x [112], and USP29 [113] interact with cGAS to remove K48-linked ubiquitin chains, thereby stabilizing the cGAS protein.

### Phosphorylation

Phosphorylation is a key mechanism for regulating protein activity and function, primarily involving the amino acids threonine, serine, and tyrosine. Increasing evidence indicates that phosphorylation and dephosphorylation of cGAS play critical roles in regulating the cGAS-STING signaling pathway. For instance, phosphorylation of cGAS at S291 in mice or S305 in humans, likely mediated by AKT [114] or CDK1 [115], significantly inhibits cGAMP synthesis, while dephosphorylation at these sites by PP1 restores activity. B-lymphoid tyrosine kinase 2 (BLK2) phosphorylates cGAS at Y215, promoting its cytosolic retention [116]. However, DNA damage induces dephosphorylation and nuclear translocation of cGAS. To this end, DNA-PK phosphorylates cGAS at T68 and S213, inhibiting its enzymatic activity [117], whereas PCV2 promotes phosphorylation at S278, leading to ubiquitin-mediated degradation of cGAS [118]. Not all dephosphorylation events activate cGAS; for example, PPP6C dephosphorylates cGAS at S420 in mice or S435 in humans, impairing substrate binding and innate immune response [119]. In summary, phosphorylation and dephosphorylation effectively regulate cGAS enzymatic activity and/or its protein abundance, impacting downstream interferon signaling and highlighting these processes as potential therapeutic targets for various diseases.

### Acetylation

Protein acetylation is a post-translational modification where an acetyl group is covalently attached to a protein, typically at lysine residues [120]. This modification can influence protein function, stability, localization, and interactions. The lysine acetyltransferase 5 (KAT5) acetylates multiple lysines at the N-terminus of cGAS (K47, K52, K62, K83), enhancing its DNA binding ability [121]. Conversely, studies have shown that aspirin can directly acetylate cGAS at K384, K394, and K414, leading to its inactivation and the suppression of downstream immune responses mediated by cGAS [122]. HDAC3 can promote the transcription of cGAS by deacetylating P65 [123]. However, there are no reports on the deacetylation of cGAS itself. Overall, more studies are needed to elucidate the impact of dynamic acetylation and deacetylation on the cGAS-STING signaling pathway and innate immune function.

### SUMOylation

SUMOylation is a post-translational modification where Small Ubiquitin-like Modifier (SUMO) proteins are attached to lysine residues on target proteins. This modification affects protein function, stability, localization, and interactions. SUMOylation is important for several processes such as nuclear transport, transcription regulation, DNA repair, and signal transduction, helping cells respond to stress and maintain balance. TRIM38 mediates SUMOylation of cGAS at K217 and K464, preventing its polyubiquitination and degradation [124]. Additionally, during viral infection, TRIM38 also SUMOylates STING, promoting its activation and protein stability [124]. Limited studies suggest that SUMOylation at different sites on cGAS has distinct functions. For example, SUMOylation of cGAS can be removed by SENP2, leading to cGAS degradation [124]. Conversely, SENP7 removes SUMOylation at cGAS K335 and K372, thereby activating cGAS [125].

### Glutamylation

Glutamylation is an ATP-dependent protein modification that attaches glutamate chains to specific glutamate residues on target proteins. This process is facilitated by tubulin tyrosine ligase (TTL) and tubulin tyrosine ligase-like (TTLL) enzymes [126]. Cytosolic carboxypeptidases (CCPs) can remove these glutamate chains. TTLL4 mediates monoglutamylation of cGAS at E302, inhibiting its synthase activity, while TTLL6 catalyzes polyglutamylation at E272, hindering its ability to bind DNA. Conversely, CCP5 removes monoglutamylation, and CCP6 removes polyglutamylation [127]. During HSV infection, TTLL4 and TTLL6 protein levels are persistently decreased, suggesting that viral infection may trigger regulatory mechanisms affecting these enzymes. Further research is needed to explore these mechanisms.

### ISGylation

ISGylation is a post-translational modification where ISG15, a ubiquitin-like protein, is covalently attached to target proteins. ISGylation can modulate protein stability, activity, localization, and interactions, playing a crucial role in antiviral responses and immune regulation [128–130]. A recent study has found that HERC5 catalyzes ISGylation of cytosolic cGAS at K21, K187, K219, and K458 in response to exogenous DNA stimulation [131]. This modification promotes cGAS oligomerization, ultimately enhancing the cGAS-STING antiviral immune response [131].

### Palmitoylation

Palmitoylation is a reversible post-translational modification where a palmitoyl group (a 16-carbon fatty acid) is covalently attached to cysteine residues of target proteins via a thioester bond. This modification affects protein membrane localization, stability, trafficking, and function.

ZDHHC18 mediates the palmitoylation of cGAS at C474, which inhibits its dimerization and thereby suppresses its activity [132]. In addition, our collaborative research discovered that ZDHHC9 mediates the palmitoylation of cGAS at C403 and C404, promoting its dimerization and activation [133]. Additionally, lysophospholipase-like 1 (LYPLAL1) can depalmitoylate cGAS, inhibiting its activation [133]. As such, targeting LYPLAL1-mediated cGAS depalmitoylation may enhance cGAS activation, offering a potential strategy to boost anti-tumor immunotherapy efficacy. Studies suggest that palmitoylation is crucial for cGAS dimerization and activation, with different enzymes mediating distinct functions at various sites. This indicates the potential for combining cGAS palmitoylation targeting with immunotherapy to explore new therapeutic possibilities.

### PARylation

PARylation is a post-translational modification where ADP-ribose polymers are attached to target proteins. PARylation plays a critical role in various cellular processes, including DNA repair, chromatin remodeling, transcription, and cell death [134, 135]. PARP1 mediates PARylation of human cGAS at D191 or mouse cGAS at E176, which blocks its ability to bind DNA and inhibits the antiviral immune response [136].

### Methylation

Protein arginine methylation affects protein function, interactions, and localization, playing a significant role in regulating gene expression, signal transduction, RNA processing, and DNA repair. PRMT5 methylates cGAS at the R124 site, blocking its ability to bind DNA and thereby weakening its antiviral capacity [137]. Oral administration of a PRMT5 inhibitor significantly protected mice from HSV-1 infection and extended their survival [137]. Activating the cGAS/STING innate immune pathway is crucial and effective for anti-tumor immunotherapy. Our research found that PRMT1 methylates the conserved R133 residue of cGAS, preventing its dimerization and further inhibiting cGAS/STING signaling in cancer cells [86]. Notably, genetic inactivation or pharmacological inhibition of PRMT1 led to the activation of cGAS/STING-dependent DNA sensing signals, significantly enhancing the transcription of type I and II interferon response genes [86]. Additionally, PRMT1 inhibition increased the number of tumor-infiltrating lymphocytes and promoted tumor PD-L1 expression in a cGAS-dependent manner [86]. Consequently, combining PRMT1 inhibitors with anti-PD-1 antibodies *in vivo* enhanced anti-tumor therapeutic efficacy.

## cGAS in Cancer

The role of cGAS in tumors has been extensively studied, revealing its dual function in both promoting and inhibiting tumor progression.

### Tumor suppressive functions of cGAS

#### Immune response enhancement:

cGAS primarily functions in the immune response by activating the downstream STING-TBK1 signaling cascade, leading to upregulation of the type I interferons. Deficiencies in the cGAS-STING pathway reduce tumor immunogenicity, lowering the efficacy of immune checkpoint inhibitors. For instance, a study on the B16F10 melanoma model showed no tumor growth difference in untreated mice with different genotypes. However, following anti-PD-L1 antibody treatment, the tumor volume in WT mice was significantly reduced, while *cGAS*-deficient mice showed no significant change [138]. Additionally, reduced cGAS expression correlates with lower survival rates in patients with invasive ductal carcinoma [139].

#### Inducing cellular senescence and clearance:

cGAS inhibits tumorigenesis and progression by promoting tumor cell senescence and clearance. For example, *cGAS* knockout MEFs exhibits reduced senescence phenotypes compared to WT MEFs [140]. Similarly, *cGAS* knockout in B16F10 cells resulted in fewer senescent phenotypes [141].

#### Autophagy and apoptosis:

cGAS also suppresses tumors by inducing autophagy and apoptosis in tumor cells [142–145].

Furthermore, some tumors may exploit mechanisms to inhibit the cGAS-STING pathway, thereby evading immune detection and promoting tumor progression. It has been reported that the cGAS-STING pathway is often suppressed in lung adenocarcinoma, colorectal cancer, melanoma, liver cancer, gastric cancer, and telomerase-deficient cancer cells [141,146–150].

### Tumor promotive functions of cGAS

cGAS can also promote tumor progression through both STING-dependent and STING-independent mechanisms.

#### STING-dependent pathways:

In MC38 colon tumors and human squamous cell carcinoma, recruitment of regulatory T cells and mobilization of myeloid-derived suppressor cells by the cGAS-STING pathway leads to reduced tumor immunogenicity [151]. In Lewis lung carcinoma (LLC), cGAS-STING promotes immune tolerance in part via activation of indoleamine 2,3-dioxygenase (IDO), supporting LLC cell proliferation [152]. Cancer cells can transfer cGAMP to astrocytes via protocadherin seven, activating the STING pathway and forming gap junctions in the brain, promoting tumor growth and metastasis [153].

#### STING-independent pathways:

cGAS acts as an inhibitor of homologous recombination DNA repair by affecting the DNA repair functions of PARP1 and RAD51, thereby compromising genomic stability [116,154,155].

Given these complexities, targeting the cGAS-STING pathway in cancer therapy involves carefully modulating its activity to enhance anti-tumor immunity while minimizing potential pro-tumorigenic effects. Research into PTMs of cGAS, such as methylation, ubiquitination, and phosphorylation, provides further insight into how the activity of cGAS can be fine-tuned to achieve optimal therapeutic outcomes [156,157].

## Targeting PRMT1-cGAS-STING Pathway for Anti-Tumor Immunity

**Figure 5.**
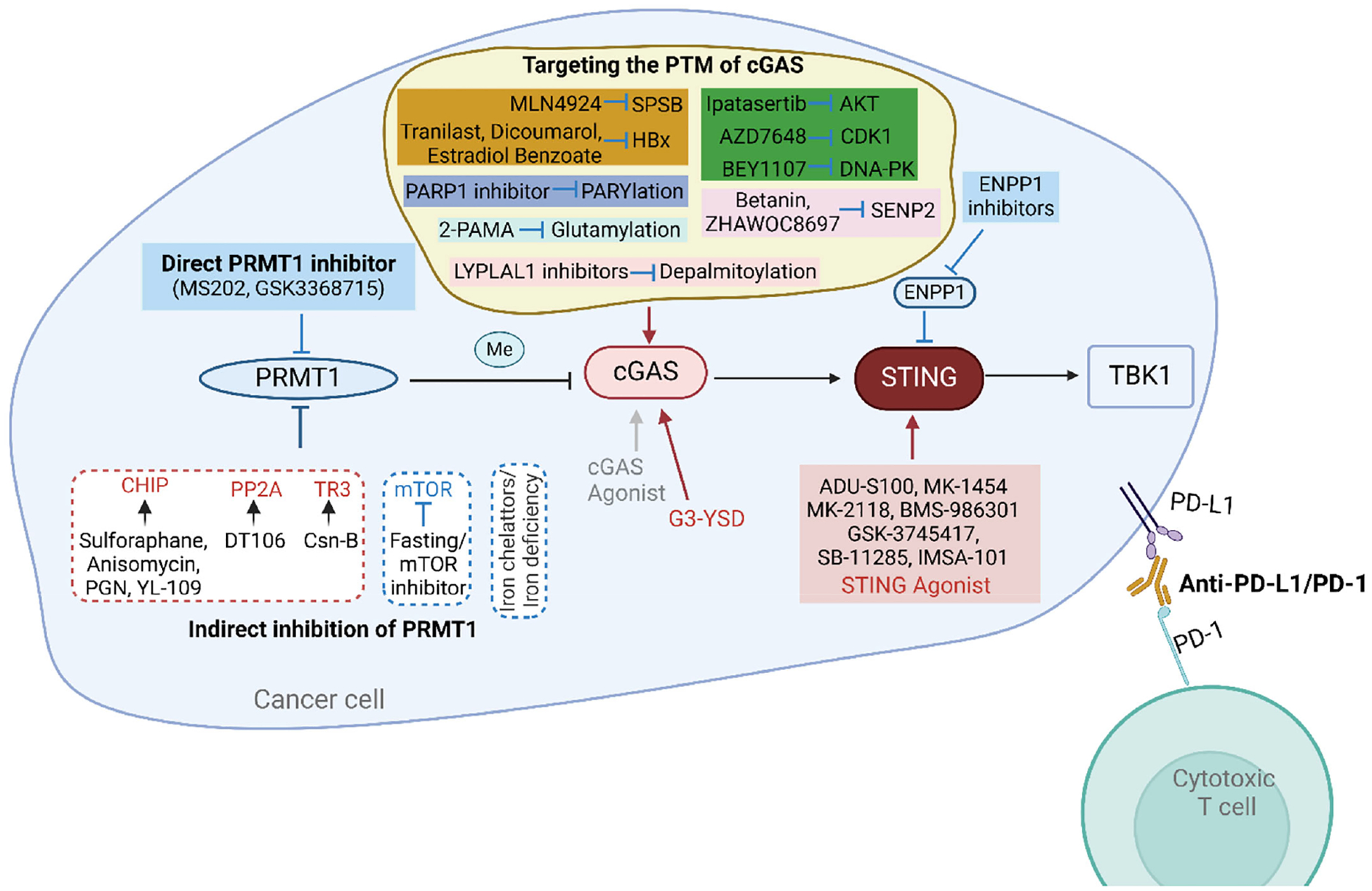
Strategies for targeting the PRMT1-cGAS-STING pathway for augmenting anti-tumor immunity.

The PRMT1-cGAS-STING pathway is a critical signaling axis in the innate immune response and has significant potential in enhancing anti-tumor immunity. The following strategies can be employed to target this pathway (Figure 5).

### PRMT1 inhibition

#### PRMT1 inhibitors:

Due to the significant role of PRMT1 in various tumors, it is a viable and worthy drug target for research. Our studies have demonstrated that using existing PRMT1 inhibitors, MS203 and GSK3368715, can activate the expression of type I and II interferon response genes and promote cGAS-dependent immune cell infiltration [86]. Previous research indicates that inhibiting both PRMT1 and PRMT5 can prevent the methylation of cGAS, thereby activating the cGAS-STING pathway and enhancing its downstream immune response [86,137]. Both PRMT1 and PRMT5 are Type I PRMTs, and inhibitors like MS203 and GSK3368715 can restore cGAS activity by simultaneously inhibiting PRMT1 and PRMT5 [86]. Therefore, pharmacological inhibition of PRMT1 is an effective strategy to activate cGAS and enhance anti-tumor immunity. The development of potent and specific PRMT1 inhibitors remains a promising area for research.

#### Search and development of autoimmune antibodies to PRMT1:

Additionally, exploring whether there are autoantibodies against PRMT1 *in vivo* or developing monoclonal antibodies against PRMT1 could be potential strategies to activate cGAS-mediated immune responses.

#### Indirect inhibition of PRMT1:

Based on the previously summarized regulatory mechanisms of PRMT1, we can aim to inhibit PRMT1 by upregulating or activating its negative regulators and downregulating or inhibiting its positive regulators. Among the numerous negative regulators of PRMT1, CHIP, PP2A, and TR3 have known agonists that enhance their inhibitory effects on PRMT1 activity or protein levels.

Specifically, four known CHIP agonists—Sulforaphane, Anisomycin, Peptidoglycan (PGN), and 2-(4-hydroxy-3-methoxyphenyl)-benzothiazole (YL-109)—induce CHIP protein overexpression and can serve as potential small-molecule inhibitors of PRMT1 [158]. The PP2A activator DT-061 inhibits tumor progression through various mechanisms, suggesting its potential to inhibit PRMT1 as part of its tumor-suppressing actions [159,160]. Thus, PP2A-activating ligands could be explored as indirect inhibitors of PRMT1 [161]. Cytochrome B (Csn-B), an agonist of TR3 [90], may suppress PRMT1 levels. Furthermore, iron chelators and iron deficiency can effectively reduce PRMT1 levels. Conversely, inhibitors of mTOR and other suppressive measures could also serve as methods to inhibit PRMT1.

### Activation of the cGAS-STING-TBK1 pathway

Many types of cancer can induce spontaneous adaptive T-cell responses and promote an immunosuppressive microenvironment that favors tumor progression. Therefore, targeting the cGAS-STING-TBK1 pathway with agonists to “heat up” the tumor microenvironment by secreting interferons and other cytokines can enhance anti-tumor immune responses.

#### Direct activation of cGAS-STING-TBK1 components:

To enhance antitumor immune effects by activating the cGAS-STING-TBK1 signaling pathway, we can use agonists to directly activate each component. Although developing activators for cGAS and TBK1 is reasonable and potentially useful, the focus has mainly been on STING agonists.

In recent years, there has been rapid progress in developing CDN analogs or non-nucleotide small molecules as STING agonists to mimic the function of endogenous 2’,3’-cGAMP [162]. Several compounds (e.g., ADU-S100, MK-1454, MK-2118, BMS-986301, GSK-3745417, SB-11285, IMSA-101) that activate STING are already in clinical studies [162]. However, agonists specifically targeting cGAS and TBK1 are rare. One study showed that G-ended Y-form DNA (G3-YSD) can interact with cGAS to activate the cGAS-STING signaling pathway rather than the RIG-I-MVAS signaling pathway.

#### Indirect activation of cGAS-STING-TBK1 components:

We previously found that cGAS activity and expression levels are regulated by various PTMs. Therefore, targeting these PTMs can indirectly activate cGAS. Inhibitors of different E3 ubiquitin ligases for cGAS can serve as crucial means to activate cGAS by preventing ubiquitination-mediated degradation and activity inhibition. For example, the neddylation inhibitor MLN4924 [163] can indirectly activate cGAS by inhibiting CUL5-SPSB3, thereby increasing nuclear cGAS levels. However, due to its broad impact on substrates, MLN4924 might also affect cellular homeostasis significantly. Several known HBx inhibitors and negative regulators, such as tranilast [164], Dicoumarol [165], and Estradiol Benzoate [166], can serve as means to indirectly activate cGAS. Given the impact of cGAS phosphorylation on its function, inhibitors of AKT1 (ipatasertib) [167], CDK1 (BEY1107) [168], or DNA-PK (AZD7648) [169] can also be used to reactivate cGAS. Using SENP2 inhibitors (Betanin [170], ZHAWOC8697 [171]) may activate cGAS by increasing SUMOylation at K217. The inhibitor 2-phosphonomethylpentanedioic acid (2-PMPA) [172] might activate cGAS by increasing glutamylation. Additionally, PARP1 inhibitors may enhance cGAS DNA-binding ability by inhibiting PARylation at D191. Inhibitors of LYPLAL1 [173] can activate cGAS by preventing depalmitoylation. PRMT1 and PRMT5 inhibitors can restore cGAS activity by inhibiting methylation at different sites, thus boosting antitumor immune responses [86, 137].

Although cGAS undergoes acetylation, SUMOylation, and ISGylation, there are currently no suitable compounds or drugs known to activate cGAS by regulating these modifications.

In addition to directly activating STING, inhibiting the phosphodiesterase ENPP1, a key negative regulator of the STING pathway [174], is another attractive method to enhance STING signaling controllably in certain tumor models. Currently, several small molecules claimed to be orally active ENPP1 inhibitors are entering clinical trials [175–177]. Similarly, targeting the PTMs of STING and TBK1 to indirectly activate this pathway is also an effective strategy; however, this will not be discussed here.

### Combination therapy

Since PRMT1 inhibits the cGAS-STING signaling pathway in tumors, methods to directly or indirectly restore cGAS activity and levels can enhance the anti-tumor immune response of cancer cells. Targeting different immune checkpoint pathways (PD-1/PD-L1, CTLA-4) can more effectively block the inhibitory signals used by cancer cells to evade the immune system, thereby enhancing T cell-mediated killing of tumor cells. Our research has found that both inhibitors and or knockdown of PRMT1 can increase PD-L1 expression and significantly improve the anti-PD1 therapy in various mouse tumor models [86]. However, the effects of combining PRMT1 inhibitors with other immune checkpoint inhibitors such as CTLA-4, LAG-3, TIM-3, and TIGIT remain to be determined. Additionally, STING agonist therapy combined with PD-1 immune checkpoint blockade has been shown to enhance the response of high-grade serous ovarian cancer patients to carboplatin chemotherapy [178] and is being evaluated in preclinical models for lymphoma [179]. Moreover, indirect inhibition of PRMT1, as well as targeting the PTM of cGAS, can be combined with each other or with immune checkpoint blockade for effective strategy to enhance the anti-tumor immunity of cancer cells.

## Conclusion

The central objective of tumor immunotherapy is to enhance the anti-tumor immunity of cancer cells and facilitate T cell-mediated killing. Activation of innate immune pathways within tumor cells, such as the cGAS-STING and RIG-I-MAVS signaling pathways, is essential to achieve this goal and improve overall anti-tumor immunity. In this review, we focused on the PRMT1-cGAS-STING signaling pathway, detailing the roles of PRMT1 and cGAS, and their PTMs, along with new strategies to activate this pathway, directly or indirectly. Recent studies indicate that targeting this pathway can effectively activate IFN signaling in tumor cells, enhance immune responses, and synergize with PD-1 to improve anti-tumor effects. However, it is necessary to investigate the efficacy of other compounds or drugs that directly activate this pathway or target upstream PTM regulators in combination with PD-1 antibodies to determine their impact on the anti-tumor immune response of tumor cells. This suggests that targeting the PRMT1-cGAS-STING signaling pathway, in conjunction with immune checkpoint inhibitors, is a potentially effective strategy in tumor immunotherapy.

## Figures and Tables

**Table 1.** Summary of PRMT1-mediated arginine methylation events and its impacts on various physiological processes.

Substrate	Methylation Residues	Biological outcomes	Reference
H4	R3	Facilitates β-globin transcription	[24]
H4	R3	Modulation of cellular senescence	[20]
H4	R3	Establishment or maintenance chromatin modifications	[37]
H4	R3	Spermatogonial development	[22]
53BP1	R1400, 1401, 1403	53BP1 DNA binding activity	[26]
53BP1	Unknown	Facilitates efficient DNA repair	[25]
MRE11	Unknown	Facilitates efficient DNA repair	[25]
MRE11	R570, 594	DNA damage checkpoint control	[27]
RUNX1	R206, 210	Abrogates SIN3A binding and potentiates its transcriptional activity	[28]
STAT1	R31	Modulates IFNα/β-Induced transcription	[29]
MyoD	R121	Activates myogenin transcription	[30]
TAF15	R203, 525, 532, R567	Positive gene regulatory function	[31]
NGN3	R65	Pancreatic endocrine development of hESCs	[33]
METTL14	R255	Promotes m6A and mESC endoderm differentiation	[38]
Klf4	R396	Restrains the commitment of primitive endoderm	[39]
Eya1	Unknown	Muscle stem cell fate	[23]
ASK1	R78, 90	Regulation of stress-induced signaling	[40]
BAD	R94, 96	Inhibits Akt-dependent survival signaling	[41]
PGC-1α	R665, 667, 669	Activation of PGC-1α	[42]
ERa	R260	Regulation of Estrogen Rapid Signaling	[43]
EGFR	R198, 200	Regulates signaling and cetuximab response	[44]
MICU1	R455	determines the UCP2/3 dependency of mitochondrial Ca2^+^ uptake	[45]
FOXP3	R48, 51	Modulate regulatory T cell functions	[34]
ILF3	R609	Induces M2 polarization of macrophages	[35]
IDH2	R353	Increasing B cell proliferation and antibody production	[46]
PFKFB3	Unknown	Promotes glycolysis and ensures stress hematopoiesis	[47]
FoxO1	Unknown	Hepatic glucose production	[48]
ATXN2L	Unknown	Controls ATXN2L localization	[32]
NPRL2	R78	Govern mTORC1 methionine sensing	[36]
UBAP2L	Unknown	Stress granule assembly	[49]
SCYL1	R787, 805	Golgi morphogenesis	[50]
RBM15	R578	RNA splicing	[51]

**Table 2.** Summary of PRMT1-mediated arginine methylation on specific downstream substrates in various types of human cancers.

Cancer type	Substrate	Methylation Residues	Biological outcomes	Reference
Breast	H4	R3	Modulates epithelial mesenchymal transition and cellular senescence	[52]
	SRSF1	R93, 97, 109	Promotes oncogenic exon inclusion events and breast tumorigenesis	[60]
	PR	R637	Promotes cells proliferation and metastasis	[58]
	PGHDH	R20, 54	Drives chemoresistance in triple-negative breast cancer	[59]
	EZH2	R342	Promotes cells proliferation, tumorigenesis, and metastasis	[55–57]
	C/EBPα	R35, 156, 165	Promotes cancer cells proliferation	[54]
	BRCA1		Defense cells against ionizing radiation	[53]
Lung	TWIST1	R34	Regulates EMT	[62]
	SOX2	R90, 98, 113, 115	Induces chemoresistance	[64]
Lung	PKP2	R101	Participates in radiation resistance	[63]
	cGAS	R127	Stabilize cGAS and promotes NSCLC cell proliferation	[61]
Colorectal	H4	R3	Enhances SLC7A11 promoter activity, promotes tumor progression	[65]
	PGK1	R206	Promotes colorectal cancer glycolysis and tumorigenesis	[66]
	RIP3	R486	Reverts the immune escape of necroptotic colon cancer	[67]
Osteosarcoma	STAT3	R688	Promotes tumorigenesis and progression	[70]
Liver	PHGDH	R213	Promotes serine synthesis and represents a therapeutic vulnerability	[69]
Glioma	H4	R3	Drives PTX3 and regulates ferritinophagy	[71]
Thyroid	H4	R3	Upregulates ZEB1 and accelerates cell proliferation, migration, and tumor growth	[80]
Pancreatic	P14^ARF^	R96, 98	Promotes the tumor suppressor function of P14^ARF^	[81]
	GLI1	R597	Increases oncogenic ability	[72]
Leukemia	FLT3	R972, 973	Promotes AML maintenance, disrupts maintenance of MLL-rearranged acute lymphoblastic leukemia	[73,74]
	WTAP	R272	Promotes multiple myeloma tumorigenesis	[75]
	H4	R3	Inhibits ACSL1 expression and ferroptosis	[76]
Ovarian	FAM98A	Unknown	Promotes cancer progression	[77]
	BRD4	R179, 181, 183	Regulates BRD4 phosphorylation and promotes ovarian cancer invasion	[78]
Gastric	c-FOS	R287	PRMT1-mediated c-FOS protein stabilization Promotes gastric tumorigenesis	[79]
	INCENP	R887	Promotes mitosis of cancer cells	[82]
	METTL14	R442, 445	Regulates m6A and promotes tumorigenesis	[68]

**Table 3.** Summary of the upstream regulators of PRMT1.

Regulator	Positive	Negative	Function	Reference
E4B	−	+	Promotes PRMT1 degradation	[92]
CHIP	−	+	Promotes PRMT1 degradation	[92]
PP2A	−	+	Inhibits PRMT1 enzymatic activity	[89]
TRIM48	−	+	Promotes PRMT1 degradation	[94]
Deferoxamine (DFO)	−	+	Down-regulates PRMT1 levels	[95]
TR3	−	+	Inhibits PRMT1 enzymatic activity	[90]
miR-503	−	+	Reduced PRMT1 mRNA levels	[96]
hCAF1	−	+	Inhibits PRMT1 enzymatic activity	[91]
BTG1	+	−	Promotes enzymatic activity	[88]
mTOR	+	−	Promotes PRMT1 expression	[87]

**Table 4.** Summary of the post-translational modifications (PTMs) of cGAS.

PTM type	Sites	Mediator	Function	Reference
Ubiquitination	K427, 428	SPSB3	Targets nuclear cGAS for degradation	[103]
	Unknown	HBx	Promotes ubiquitination and autophagy degradation	[102]
	Unknown	TRAF6	Activates cGAS signaling	[104]
	K173, 384	RNF185	Promotes cGAS activity	[105]
	Unknown	TRIM41	Promotes cGAS activity	[106]
	K335	TRIM56	Increases DNA-binding activity	[107]
	K411	RNF178	Inhibits the DNA binding ability	[108]
Deubiquitination	K414	USP14	Stabilizes cGAS	[111]
	Unknown	USP27x	Stabilizes cGAS	[112]
	K271	USP29	Stabilizes cGAS	[113]
Phosphorylation	S291 (m), S305 (h)	AKT	Inhibits cGAS activity	[114]
	Y215	BLK2	Facilitates the cytosolic retention of cGAS	[116]
	S291 (m), S305 (h)	CDK1	Inhibits cGAS activity	[115]
	T68, S213	DNA-PK	Inhibits cGAS activity	[117]
	S278	PCV2	Inhibits cGAS activity	[118]
Dephosphorylation	S291 (m)	PP1	Promotes cGAS activity	[115]
	S420 (m), S435 (h)	PPP6C	Inhibits cGAS activity	[119]
Acetylation	K47, 52, 62, 83	KAT5	Increases DNA-binding activity	[121]
	K384, 394, 414	Aspirin	Blocks cGAS Activity	[122]
SUMOylation	K217 (m), K464 (m)	TRIM38	Prevents cGAS degradation	[124]
DeSUMOylation	K217 (m), K464 (m)	SENP2	Promotes cGAS degradation	[124]
	K335 (m), K372 (m), K382 (m)	SENP7	Potentiates cGAS activation	[125]
Methylation	R124	PRMT5	Blocks the DNA binding ability	[137]
	R133	PRMT1	Prevents cGAS dimerization	[86]
Glutamylation	E302 (m)	TTLL4	Promotes cGAS activity	[127]
	E272 (m)	TTLL6	Promotes cGAS activity	[127]
Deglutamylation	E302 (m)	CCP5	Inhibits cGAS activity	[127]
	E272 (m)	CCP6	Inhibits cGAS activity	[127]
ISGylation	K21, 187, 219, 458	HERC5	Facilitates cGAS oligomerization	[131]
Palmitoylation	C474	ZDHHC18	Negatively regulateS cGAS activation	[132]
	C404, 405	ZDHHC9	Facilitates cGAS dimerization and activation	[133]
Depalmitoylation	C404, 405	LYPLAL1	Inhibits cGAS activity	[133]
PARylation	D191	PARP1	Inhibits DNA binding ability	[136]
